# *Cowpox virus*: What’s in a Name?

**DOI:** 10.3390/v9050101

**Published:** 2017-05-09

**Authors:** Matthew R. Mauldin, Markus Antwerpen, Ginny L. Emerson, Yu Li, Gudrun Zoeller, Darin S. Carroll, Hermann Meyer

**Affiliations:** 1 Poxvirus and Rabies Branch, Centers for Disease Control and Prevention, 1600 Clifton Road NE, Atlanta, GA 30333, USA; GEmerson@cdc.gov (G.L.E.); YuLi@cdc.gov (Y.L.); DCarroll@cdc.gov (D.S.C.); 2 Oak Ridge Institute for Science and Education, P.O. Box 117, Oak Ridge, TN 37831, USA; 3 Bundeswehr Institute of Microbiology, Neuherbergstr 11, 80937 Munich, Germany; MarkusAntwerpen@bundeswehr.org (M.A.); gudrunzoeller@bundeswehr.org (G.Z.); hermann1meyer@bundeswehr.org (H.M.)

**Keywords:** *Cowpox virus*, smallpox, *Vaccinia virus*, poxvirus, phylogenomics, monophyly, polyphyly, species delimitation, taxonomy, misnomers

## Abstract

Traditionally, virus taxonomy relied on phenotypic properties; however, a sequence-based virus taxonomy has become essential since the recent requirement of a species to exhibit monophyly. The species *Cowpox virus* has failed to meet this requirement, necessitating a reexamination of this species. Here, we report the genomic sequences of nine *Cowpox viruses* and, by combining them with the available data of 37 additional genomes, confirm polyphyly of *Cowpox viruses* and find statistical support based on genetic data for more than a dozen species. These results are discussed in light of the current International Committee on Taxonomy of Viruses species definition, as well as immediate and future implications for poxvirus taxonomic classification schemes. Data support the recognition of five monophyletic clades of *Cowpox viruses* as valid species.

## 1. Introduction

This study examined a group of non-cellular organisms, which consist of proteins and genetic material. These organisms are capable of invading and replicating within living cells. The preceding sentences would have been more concise if the name of these organisms (viruses) had been used instead of a description. We often use names to facilitate the communication or categorization of topics or objects; however, names only assist communication when all parties utilize the same moniker. Unfortunately, common names vary between languages and regions. In one example from the southeastern United States, both pocket gophers (fossorial mammals) and salamanders (amphibians) are frequently referred to as salamanders [1,2].

Fortunately, the hierarchical nature of scientific names, along with the disallowance for multiple organisms to use the same official name, can drastically reduce these potential miscommunications. The International Congress on Taxonomy of Viruses (ICTV; [3]) is the governing body for recognizing official names of viruses, and defines a species as “a monophyletic group of viruses whose properties can be distinguished from those of other species by multiple criteria” [4]. This recent definition change has placed more emphasis on common descent, which should lead to a better understanding of the true diversity and the evolutionary history of viruses [5]. These new guidelines for species recognition will have implications not only for newly discovered virus species, but also for currently and historically recognized species.

One example of an impacted group of viruses is the genus *Orthopoxvirus* (*Poxviridae*: *Chordopoxvirinae*). This genus contains 10 currently recognized species, including four which are pathogenic to humans: *Variola virus* (VARV), the causative agent of smallpox, *Monkeypox virus* (MPXV), *Vaccinia virus* (VACV), and *Cowpox virus* (CPXV) [6,7] as well as species which are of veterinary importance: *Mousepox virus* (ECTV) and *Camelpox virus* (CMLV). Initially, prior to the concept of viruses, the disease smallpox was known as the “pox”, later the term ‘smallpox’ was used to distinguish it from the ‘great pox’, i.e., syphilis [8]. Likewise, local lesions on the teats of cows were referred to as ‘cow-pox’. It was Edward Jenner who identified and differentiated two clinical entities as “true cow-pox” and “spurious cow-pox” [9]. He noticed that only an infection with “true cow-pox” protected milk maids during smallpox outbreaks [10]. In 1937, the causative agent of cowpox was isolated from a lesion on a milker’s hand. At that time, outbreaks in cows were commonly reported across Europe [11]. It was the accepted practice of naming a virus after the sick or diseased animal from which it was first isolated. In 1971 the first report of the ICNV (later the ICTV) set a standard in viral taxonomy and a total of 290 virus species, causing distinct diseases, were recognized, 34 of which belonged to the Genus *Poxvirus* [12]. Ten virus species listed (*Alastrim virus*, *Buffalo pox virus*, *Camelpox virus*, *Cowpox virus*, *Horse pox virus*, *Infectious ectromelia virus*, *Monkeypox virus*, *Rabbitpox virus*, *Vaccinia virus* and *Variola virus*) were later assigned to the Genus *Orthopoxvirus* [13].

Baxby [14] analyzed 17 *Cowpox virus* strains from humans and cows and demonstrated a good deal of variability in certain biological characteristics (including mouse virulence), but all produced red hemorrhagic pocks on the chorioallantoic membrane and A-type inclusion bodies. Over the years, the number of cases of bovine cowpox decreased, and increasing numbers of clinically similar infections were reported in cats and elephants which led to their classification as catpox virus and elephantpox virus. In addition, viruses were isolated from various zoo animals (cheetah, jaguarundi, lion, panther, beaver, marmoset, mongoose, rats, etc.) [15,16,17] and the term “cowpox-like viruses” was used to classify them. However, all these viruses induced red hemorrhagic pocks on the chorioallantoic membrane and A-type inclusion bodies [18,19,20], thus, they were considered as belonging to a single species, *Cowpox virus*. With the last case of cowpox in a cow reported more than a quarter century ago [21], it is generally accepted that *Cowpox virus* is misnamed [22]. Rodents are the primary reservoir for *Cowpox virus*, with various namesake animals (and humans) being accidental hosts of a chain of transmission [18,23]. Given the knowledge gained over the last half century (i.e., an understanding of the broad host range and the frequency with which these accidental hosts are infected), it has become apparent that naming a virus after the first organism to be infected can (and has) resulted in misnomers [22], with CPXV being a prime example, as well as the focus of this study.

Attempts to determine the genotype of orthopoxvirus (OPXV) species by the generation of Restriction Fragment Length Polymorphisms (RFLP) data confirmed the species concept originally based on phenotypic characteristics. By applying RFLP, it became evident that both buffalopox and rabbitpox virus indeed belonged to the species *Vaccinia virus*. In contrast, analyzing multiple isolates from cows, humans, elephants and cats demonstrated a much larger degree of variability among these CPXV strains as compared to other species of the genus *Orthopoxvirus* [21,24]. By using Sanger sequencing and Next-Generation DNA sequencing platforms it became evident that CPXV isolates consistently failed to form a single monophyletic clade and the number of independent lineages grew as taxonomic sampling increased [25,26,27,28,29,30,31]; however, these new data did not violate ICTV rules until the species definition was changed in 2013.

Furthermore, although the ICTV is the governing body for recognizing names of viruses and has provided requirements that must be fulfilled for a given species to be officially recognized (monophyly and differentiability), there is no set of ICTV-sanctioned species delimitation criteria, such as thresholds of nucleotide/amino acid sequence (e.g., genetic variation or divergence between lineages). Since given rates of evolution between RNA and DNA viruses vary drastically, the ICTV has refrained from positing species delimitation guidelines. Subsequently, lineage-specific working groups have recommended specific criteria for discriminating and delineating species or strains [32,33,34,35]. Some of these working groups have set a divergence threshold based on amino acid or nucleotide data, which must be met for an isolate to represent a new species (*Filovirus*; *Arenavirus*), but have not taken intraspecific genetic variation into consideration. Given the nature of diversification, the amount of intraspecific genetic variation present within lineages should be considered when determining the extent of inter-lineage genetic variation necessary to warrant species level recognition [5,36,37,38,39].

It is widely accepted that gene trees and species trees are not necessarily identical. This may be a result of incomplete lineage sorting or selection pressures (among other things), depending upon the study organism. Phylogenetic analyses of poxviruses have generated highly-supported, yet contradictory topologies depending upon the genome region examined [26,28]. Therefore, when considering genetic variation in poxviruses, it is necessary to determine which genomic region(s) (i.e., one gene, multiple genes, conserved coding region, entire coding region, whole genome including inverted terminal repeats) should be examined to provide the ‘true’ evolutionary relationships. Some authors have utilized the entire coding region excluding inverted terminal repeats [27], whereas others have examined a smaller, more conserved central region of the genome [26], and yet others have analyzed various subsets of protein coding genes [28,30,40]. Each potential dataset’s pros and cons should be considered from a biological perspective to determine if any phylogenetic assumptions might be violated. Genetic distance calculations between isolates and species are expected to decrease when comparing whole genomes, conserved central regions, or concatenated open reading frame datasets; however, utilizing sequences further from the variable termini should reduce any expectation of recombination issues.

Some researchers [26,27,28] have noted the polyphyly of *Cowpox viruses*, but have not set forth recommendations regarding how to split the species into a number of monophyletic species to conform to the ICTV regulations. Additionally, no researchers to date have examined the species *Cowpox virus* using statistical delimitation tools. Given these data, the purpose of this research was to: (1) increase the geographic sampling of CPXV genomes; (2) conduct genomic level phylogenetic analyses; (3) use a coalescent approach to run species tree and species delimitation analyses; (4) consider the validity of the species *Cowpox virus* as currently understood given the new ICTV definition of a virus species; and (5) determine if variability in the dataset (genome region) examined has the potential to affect the monophyly of *Cowpox virus* lineages.

## 2. Materials and Methods

### 2.1. Culturing, Sequencing, Assembly, and Annotation

Whole genome nucleotide sequences from nine CPXV isolates collected from localities throughout the United Kingdom (UK) and Norway were generated for this study. CPXV strains were grown on African Monkey Kidney cell line MA 104. Suspensions were freeze-thawed three times and DNA was extracted using the automated DNA extraction system MagNA Pure Compact (Roche Diagnostics, Penzberg, Germany). DNA quantification was measured using fluorometric system Qubit 2.0 (Life Technologies, Darmstadt, Germany), and purity was estimated by nanodrop (Thermo Scientific, Darmstadt, Germany).

Sequence libraries of isolated genomic DNA were prepared using TrueSeq PE library preparation kit (Illumina, San Diego, CA, USA). One nanogram of final library prep was used as input for the Illumina HiSeq 2000 system at Otogenetics (Atlanta, GA, USA). Reads were mapped to the reference genome CPXV strain Brighton Red (NC_003663.2) and variant calling was done using bowtie2 [41], samtools [42] and VarScan [43]. The Genome Annotation Transfer Utility (GATU [44]) was used for annotation of genomes. The criteria for annotation included a cut-off of at least 200 nt and 90% nt similarity with CPXV strain Brighton Red. After the transfer of annotation, assigned open reading frames of all genomes were visually inspected and corrected manually, if needed. All generated genomes were deposited into NCBI GenBank as a BioProject (PRJNA369073). Accession numbers are included in Table 1.

### 2.2. Phylogenetic Analysis

Forty-six CPXV strains (nine generated for this study) were examined along with 11 other OPXV genomes, representing all recognized species of Old World orthopoxviruses. Localities, accession numbers, and other pertinent data for all examined strains are available in Table 1. MAFFT v.7.125 [57] was used to generate the multiple sequence alignment using the FFT-NS-2 algorithm through the auto-select option. Given the concern of recombination at the variable termini [26,58], and the potential for recombination to affect the outcome of phylogenetic analyses [59], two datasets were examined for phylogenetic and species delimitation purposes. Dataset 1 (150,718 bp) included the entire coding portion of the alignment (genes C23L-B29R) with gap columns removed; whereas dataset 2 (95,410 bp) examined a smaller, conserved central region (E2L-A32L) with gap columns removed. It is worth noting the C23L-B29R alignment was identical to the whole genome alignment with gaps stripped. The most appropriate model of molecular evolution was selected with the Akaike information criterion using MrModelTest (v.2.2 [60]) and PAUP* (version 4.0b10 [61]) for each dataset. Outgroup selection was made based on phylogenetic relationships of Old World orthopoxviruses generated by previous studies and the availability of whole genome data [62,63].

Phylogenetic analyses were conducted using MrBayes v.3.2.2 [64] with the following settings: substitution model = GTR, rate variation = invgamma, gamma catagories = 4, chain length = 10 million generations, samplefreq = 1000, nruns = 2, nchains = 4, burnin = 2500, and outgroup was set to ECTV. MEGA (v.6.06 [65]) was used to calculate average pairwise distances (uncorrected p), whereas patristic distances (tree branch lengths) were calculated from the consensus tree using the program Geneious (v.7.1.7; Biomatters Inc., Aukland, New Zealand). Genetic distances were averaged across taxa to produce a single value for each inter- or intra-clade estimate. For thoroughness, a RAxML analysis starting with a completely random tree was run for each dataset using 1000 rapid bootstrap iterations followed by a search for the best scoring MLtree under the GTR + I + G model of molecular evolution using the RAxML plugin in Geneious [66].

### 2.3. Species Delimitation and Species Tree Analyses

The software Bayesian Phylogenetics and Phylogeography (BP&P v.3.1 [39,67]) was utilized to calculate statistical support simultaneously for species delimitation and species tree in a Bayesian framework. The smallest monophyletic groupings from the MrBayes topologies with divergence levels approximately equal to the divergence between *Taterapox virus* (TATV) and *Camelpox virus* were each examined for putative species level divergence. The average within species genetic distance prior, Ө, for BP&P was estimated based on two multiple sequence alignments of large genomic regions; 45 MPXV isolates examined by Nakazawa et al. [68], and 49 VARV isolates (GenBank accession numbers: L22579 [69], NC_001611 [54], Y16780 [45], DQ437580-DQ437592, and DQ441416-DQ441448 [47]) using MEGA v.6.06 [65] with pairwise deletion and uncorrected p-distance settings. Estimated values for MPXV (0.169%) and VARV (0.137%) were averaged and this value (0.153%) was used as the mean of the gamma distribution for the Ө prior. The τ prior was calculated as the average genetic distance (uncorrected p) of all in-group taxa to ECTV (3.61% and 3.01% for datasets 1 and 2, respectively). All BP&P analyses were run in duplicate with random seeds to check convergence. Each dataset was analyzed with their respective τ parameter (τ^2^ = 301[α], 10,000[β], τ^1^ = 361[α], 10,000[β]) with three Ө priors (Ө_A_ = 1[α], 654[β]; Ө_B_ = 2, 654; Ө_C_ = 2, 327), where Ө_A_ is based on the calculated average intra-species distance of MPXV and VARV, Ө_B_ is twice that average, and Ө_C_ is four times the calculated average to provide sequentially more conservative delimitation estimates. Both MrBayes topologies were used as the starting species tree topology for each dataset. Based on Bayesian inference analyses, 14 CPXV lineages were examined for consideration as putative species, and algorithm 0 was used for species delimitation with initial fine tune parameters set to 0.00019, 0.00007, 0.00339, 0.00033, 0.01521, 0.33, 1.0. All analyses were run with a burn-in of 20,000, a sample frequency of 5, and the total number of samples was set to 100,000.

### 2.4. Topology, Dataset Heterogeneity, and Recombination Testing

The incongruence length difference (ILD) test is used to determine whether two datasets (often loci) should be combined, or if they should be analyzed separately due to significant differences in signal, as measured by the length of trees generated by each dataset. In this instance, we are comparing the portion of the genome examined in dataset 2 (bp 28,069–123,478) against the two peripheral regions (bp 1–28,068 & 123,479–150,718), which, when combined, comprise dataset 1. The reason for comparing these partitions is to determine if whole genome analyses might be considered inappropriate based on some metrics. Given the variable trees generated by different datasets in this study, these partitions were used and a heuristic search with a random starting seed and 10,000 replicates was run using PAUP* (v.4.0b10) to determine if the null hypothesis of homogeneous partitions could be rejected. A topological comparison test (Kishino Hasegawa) was utilized to compare both topologies generated by MrBayes, to determine which tree showed the best congruence to each dataset. The Pairwise Homoplasy Index, Maximum χ^2^ and the Neighbor Similarity Score tests of recombination were performed using permutation tests for both datasets in the program PhiPack [70,71,72,73].

## 3. Results

### 3.1. Phylogenetic Analyses

**Figure 1:**
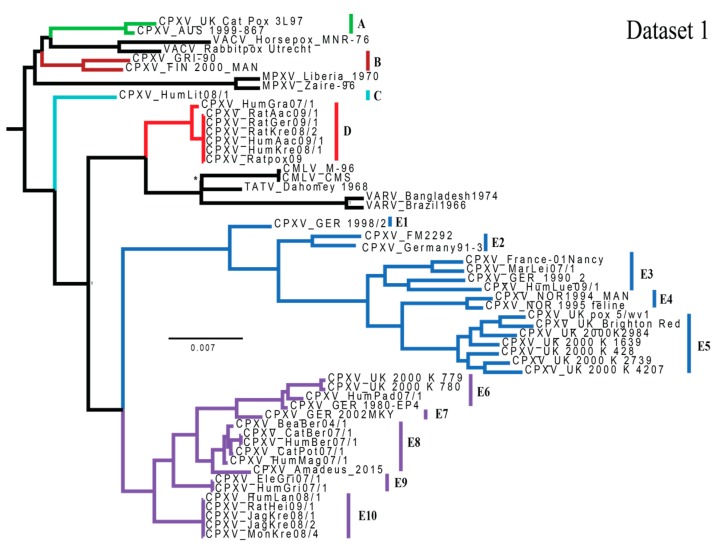
A majority-rule consensus tree generated from the MrBayes analysis of dataset 1 (150,718 bp). All Bayesian Posterior Probability (BPP) values were ≥0.95 with the exception of the Camelpox virus/Taterapox virus relationship, indicating high credibility of the branching (* BPP = 0.5181). Scale bar indicates the number of mutations per site. Clades are colored in the same manner as Dabrowski et al. [26]. Labels (A, B, C, D, E1–10) refer to monophyletic clades examined and discussed in the text. Outgroup is not pictured.

Bayesian inference analysis of dataset 1 recovered a highly-supported tree (Figure 1), with only two small polytomies (in clades D & E10) as a result of two sets of almost identical sequences, each representing a single outbreak consisting of several isolates (Table 1). All Bayesian Posterior Probability (BPP) values were ≥0.95, with the single exception of the CMLV/TATV relationship, which supports the high credibility of the overall topology. The CPXV polytomies and low support at the TATV/CMLV node have been reported in previous studies [27,28]. This topology identifies a minimum of five monophyletic clades (A–E). BP&P analyses require all putative species to be defined a priori; BP&P will not split a defined species, but can combine two or more defined species. Therefore, in subsequent analyses, clade E was further broken down to allow for the maximum resolution among the 10 subclades. Additionally, clades E1–E5 and E6–E10 were discussed as independent monophyletic clades by Dabrowski et al. [28], and are therefore discussed similarly (at times) herein.

**Figure 2:**
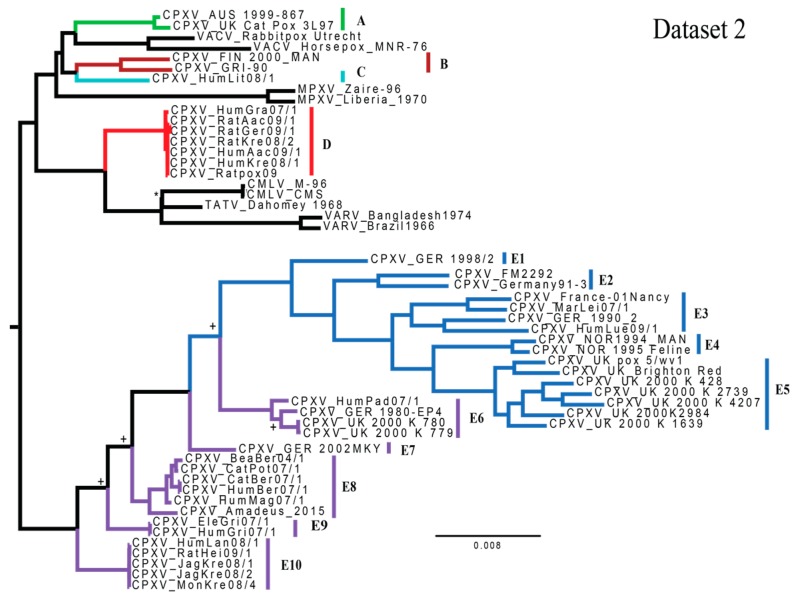
A majority-rule consensus tree generated from the MrBayes analysis of dataset 2 (95,410 bp). All BPP values were ≥0.95 unless otherwise noted by the following symbols: * (BPP = 0.5657) and ^+^ (BPP = 0.8424). Scale bar indicates the number of mutations per site. Clades are colored in the same manner as Dabrowski et al. [26]. Outgroup is not pictured.

Dataset 2 also generated a highly-supported topology (Figure 2) with five nodes calculated to have a BPP of less than 0.95 (the TATV/CMLV relationship and four nodes within clade E). This topology identifies a minimum of four monophyletic groupings of isolates (A, B/C, D & E). The most noticeable differences between these two trees are the placement of HumLit08/1 (from Lithuania; clade C) and relationships of subclades within clade E (specifically, variability in the reciprocal monophyly of E1–E5 and E6–E10). Inter-clade genetic (uncorrected p) and patristic distances for major and minor clades are available in Supplementary Tables S1 and S2, calculated for datasets 1 and 2, respectively. Each of the minor clades highlighted (E1–E10) is at least as divergent (uncorrected p-distances) from its sister clade as two currently recognized species CMLV and TATV. Intra-lineage genetic distances can be seen in Supplementary Table S3. Topologies generated using RAxML were nearly identical for each dataset, displaying similar movement of clade C and conditional polyphyly of clades E6–E10 (Supplementary Figures S1 and S2).

### 3.2. Species Delimitation and Species Tree Analyses

In all instances, regardless of dataset examined or starting tree topology, duplicate runs of the joint species tree estimation/species delimitation analyses converged on the same number of species of *Cowpox viruses* (*n* = 14); however, the species tree topologies varied, none having above 0.50 BPP. For species delimitation analyses using a fixed guide tree, all 14 examined CPXV lineages had BPP values of 0.95 or greater for each set of priors, and therefore are statistically, sufficiently divergent to warrant species level recognition.

### 3.3. Topology, Dataset Heterogeneity, and Recombination Testing

The Kishino Hasegawa test determined the tree generated for each dataset by MrBayes was significantly better for the examined dataset than the topology generated by the opposite dataset (i.e., when measuring topologies against dataset 1, Figure 1 was a significantly better fit to the data than Figure 2, but when measuring topologies against dataset 2, Figure 2 was significantly better than Figure 1). ILD test results from PAUP*, indicated a significant difference in the heterogeneity between the central and peripheral partitions (*p* = 1 × 10^−4^). Tests utilized to detect recombination (Pairwise Homoplasy Index, Maximum χ^2^ and the Neighbor Similarity Score) returned *p*-values of *p* = 0.000, indicating high levels of homoplasy, potentially explained by recombination.

## 4. Discussion

### 4.1. Phylogenetics and Species Delimitation

The CPXV genomic dataset has been expanded by the addition of nine, newly sequenced isolates from the UK and Norway. These isolates fit within the previously described clades [27,28] and do not represent any previously unidentified lineages. The sole new sequence from Norway (isolated from a cat) grouped sister to the only other Norway sequence publicly available (isolated from a human), but differed by 262 mutations (based on the conservative genomic region examined in dataset 2). The Catpox 3L97 isolate grouped sister to an isolate from Austria, which was isolated in 1999, but differs by 144 single nucleotide polymorphisms (based on dataset 2). This large geographic expanse between the only isolates of this monophyletic clade A is puzzling, and could represent the only sampled isolates from a large range, or could indicate importation of this isolate from one of the two countries to the other. The remaining isolates from the UK grouped with high support with German samples (clade E6) or other UK isolates, including the historic UK Brighton Red strain and one published by Carroll et al. [27] (clade E5). Previous to this study, the only available isolates from the UK had grouped together. This study drastically increases our understanding of the genetic variation within UK CPXV strains. The large genetic variation found in UK isolates (grouping within clades A, E5, and E6) is similar to that found in German isolates (grouping within clade D and all E subclades except E4 & E5). At this point it is unclear if these countries represent hotspots with large numbers of CPXV lineages or if this is a sampling bias issue, as more than 80% of isolates available are from either Germany (61%) or the UK (22%). Clearly, it is worth expanding geographic sampling to other areas of Eurasia to better understand the genetic diversity. At present, large areas in western, southern, and eastern Europe are not represented. It is possible that *Cowpox viruses* do not occur in these areas, but it is also possible that this paucity of geographical data is due to a sampling bias. Recent research has identified new orthopoxviruses in areas with no previous evidence of poxvirus circulation [62,63]).

Similar topological differences between datasets and the movement of the Lithuania isolate throughout the tree was also noted by Dabrowski et al. [28]. In both studies the smaller dataset placed the Lithuania strain with the ‘Vaccinia-like’ group, whereas the larger dataset placed this group sister to either clade E or the clade containing CMLV, TATV and VARV as well as CPXV clades D and E. The same study reported conditional monophyly of clades E6–E10, depending upon the dataset examined (similar to results exhibited in this study). In their larger dataset, clades E1–E5 and E6–E10 are monophyletic sister clades; however, in multiple smaller datasets Dabrowski et al. [28] recovered a paraphyletic E6–E10 with respect to clades E1–E5. The central portion of the genome is conserved and comprised of genes understood to perform essential tasks (i.e., viral replication, virion assembly and release), whereas the peripheries are more variable [74], and are thought to characterize host specificity and pathogenicity [30,75].

The issue of various genomic regions of a single replicon generating unique topologies is one worth considering. The ILD test results indicate the central region of the genome and the termini contain different evolutionary signals, hence the distinct topologies generated through analyses of both datasets. Many (if not all) phylogenetic (or population genetics) programs assume no recombination occurs within a locus, but free recombination between different loci [76,77,78,79]. Potential recombination within the variable termini of poxvirus genomes could violate this assumption and cause issues with topology generation depending upon the dataset examined and software utilized. Despite results of the analyses utilized to detect potential recombination, (*p* = 0.000 for Pairwise Homoplasy Index, Maximum χ^2^ and the Neighbor Similarity Score), these datasets were analyzed by phylogenetic inference packages for a number of reasons. First, these tests examine datasets for homoplasies, which may indicate recombination, but may also be caused by other events (convergent evolution, retention of ancestral polymorphisms, or stabilizing selection). Second, although recombination does occur in *Vaccinia virus* within laboratory settings [80], and has been suggested to occur in cowpoxviruses [81], as for it to occur in nature multiple species of viruses would need to infect the same cell of the same animal at the same time. Given the short incubation time of poxviruses, the location of viral DNA replication (cytoplasm), and the variability in both host range and geographic distributions between species, large and frequent recombination events are expected to be rare and limited in effect when analyzing the entire virus genome.

To understand the true evolutionary history of this genus, a subset of nucleotide data containing phylogenetically informative characters, capable of generating accurate topologies needs to be identified. This issue has a direct effect on the ability to delineate species, specifically when it comes to the requirement of monophyly. It may be beneficial for members of the poxvirus community to thoroughly examine and recommend a specific subset of sequence data to be used when determining if a newly sequenced isolate is representative of a new or existing species. The caveat would be that these specified data should be capable of generating consistent, and ‘accurate’ phylogenies and criteria or methodology for species delimitations should be standardized.

Phylogenetic analyses of available orthopoxvirus genomes revealed that CPXV, as currently recognized, is a polyphyletic assemblage of 5–14 highly-supported lineages based on monophyly and genetic distance criteria within a statistical framework (Figure 1). Of these evolutionarily independent lineages, five (A–E) is the smallest number of species necessary to concur with the ICTV requirement of monophyly, although this arrangement would leave a greater amount of genetic variation within clade E than in clades A–D (Supplementary Table S3). Statistical support for 14 species of CPXV was found through BPP analysis, and the examination of patristic and genetic (uncorrected p) distances indicated that each of these 14 (sub)clades are either: (1) at least as divergent as CMLV and TATV from their sister clade; or (2) group sister to a non-CPXV species. However, understanding the ‘true’ evolutionary relationships between subclades E1–E10 is necessary to determine the number of species actually represented within this grouping. Increased taxonomic sampling has the potential to solidify the relationships between these congeners.

### 4.2. Geographic Sampling

**Figure 3:**
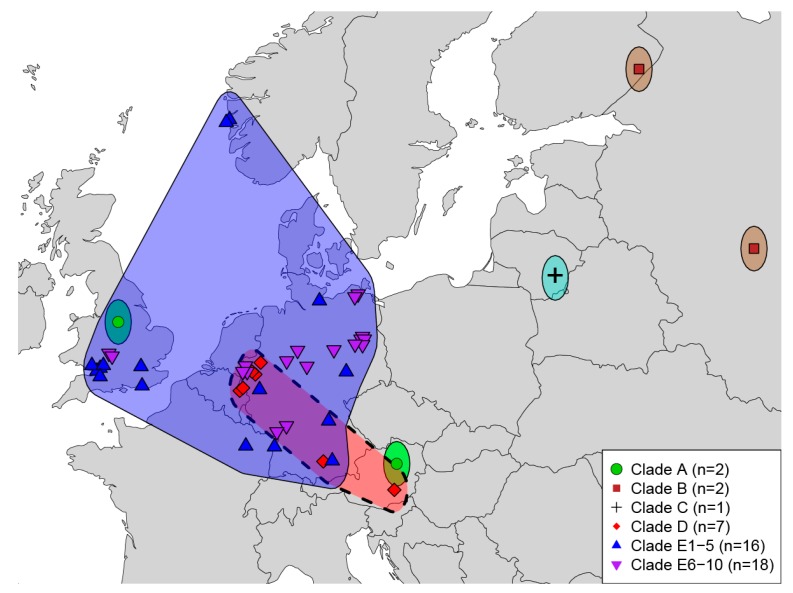
Map of cowpox virus isolates examined in this study. Polygons are present for visualization and are not intended to represent geographic ranges of viruses. The dashed polygon is indicative of a pet rat outbreak with large expected amounts of anthropogenic movement of isolates.

Geographic coverage of analyzed CPXV samples is depicted in Figure 3. Examining CPXV as currently recognized in a geographic context is puzzling, as no geographic trends are evident. The idea behind examining the phylogeographic pattern of a species or group of species assumes these organisms originate from a single most recent common ancestor (MRCA); however, numerous studies [26,27,28,29] have come to the conclusion that CXPV isolates do not all share a single MRCA. Therefore, examining each of these monophyletic clades independently would offer a more accurate picture regarding the evolutionary history and phylogeography of each clade or species. As geographic sampling has increased over the last two decades, the number of recognized monophyletic assemblages of CPXV also has increased from two [26] to three [27] to as many as 14 today. With continued sampling from human and animal cases [62,63], understanding of the genetic diversity and evolutionary relationships between orthopoxviruses is expected to improve.

### 4.3. Conclusions and Future Work

In closing, the underlying assumption of a single, most recent common ancestor shared exclusively by all representatives of CPXV has been disproven again. This time, statistical delimitation analyses have supported species level recognition of several monophyletic clades. The minimum number of monophyletic clades required to meet the ICTV requirement of monophyly within a species, when considering both potential topologies generated (as well as the robust phylogenies generated by Dobrowski et al. [28]) is five (clades A–E), although statistical support exists for up to 14 lineages. Monophyly is one part of the new species definition, the second states the properties of a species of virus “can be distinguished from those of other species by multiple criteria” [4]. This portion of the definition was likely intended to ensure valid species were not split due to inconsequential differences. The major issue with this requirement is ‘multiple criteria’ could (in theory) be any set of non-evolved traits (i.e., geographic locality, host organism). Peterson [5] provided a thorough review of this issue, and stressed the most important criteria is the evolutionary history and whether these lineages are on independent evolutionary trajectories. In keeping with the current definition of a virus, additional phenotypic, evolved traits (phenotypes derived from the genotype), such as A-type inclusions, pathogenicity, organ tropism, plaque size, incubation period, heat resistance, etc., should be examined to determine what variation may exist between and within lineages A–E.

Given the variability in topologies and the requirement for virus species to be differentiated by a number of criteria, a conservative approach in recognizing five species is proposed (clades A–E). As CPXV has been a recognized species for many decades, and referenced in the seminal works of vaccination [8,9], differentiation of the species is not trivial. To maintain continuity within the literature, it is proposed these evolutionarily independent clades be referred to as follows: *Cowpox virus alpha*, *Cowpox virus beta*, *Cowpox virus gamma*, *Cowpox virus delta*, and *Cowpox virus epsilon*. In this approach, the authors hope to minimize unnecessary changes in the literature, but still recognize the diversity within CPXV, as currently recognized. Future work may divide one or more of these species, but reducing multiple species into one will likely not be necessary.

As geographic sampling has increased over the past few decades, our understanding of the genetic diversity of *Cowpox viruses* has increased dramatically. When considering names for new taxa (isolates, species or genera), effort should be taken to propose informative names which reduce confusion (within the research community as well as the public in general) and follow nomenclatorial requirements. A systematic approach considering a variety of data should be considered when proposing names. For each species, the first published genome could be considered the type specimen and names could represent a geographic region where the virus was first discovered, or genomic or phenotypic characteristics (e.g., indels, gene synteny, incubation period, disease progression) common to members of a lineage. Both of these are accepted methods in naming other organisms (mammals); however, given the scale of global travel and variable incubation times of pathogens, naming a lineage after a geographic region could result in a misnomer if few isolates are known. *Orthopoxviruses* have been named for the animal from which initial isolates were recovered (e.g., *Monkeypox virus*, *Cowpox virus*), but further research determined these names to be misrepresentations, as the suspected primary reservoirs are now thought to be rodents. The apparent overlapping geographic and host ranges of many *Cowpox viruses* suggests some could share reservoir host species as well. The International Committee for Zoological Nomenclature has a large number of rules and regulations, which reduces confusion of recognized species names and keeps track of precedence or priority among type specimens of various lineages. To implement this number of rules in viral taxonomy would be a massive undertaking, but considering the rationale behind these rules and using this to further develop regulations for naming viruses would help with consistency of new virus names.

## Figures and Tables

**Table 1:** Table of all orthopoxvirus strains examined in the phylogenetic analyses including species, strain name, host (organism from which virus was extracted), year of isolation, GenBank accession number, locality data, clade within the MrBayes topologies, and original genome references. Isolates with identical symbols (^§^, ^Δ^, ^‡^) are from the same outbreak or are otherwise linked epidemiologically.

Species	Strain	Host	Year	Accession#	Location of Origin	Clade	Reference
CPXV	UK Cat Pox 3L97	Cat	1977	KY549143	UK	A	This paper
CPXV	AUS 1999-867	Cat	1999	HQ407377	Texing, Austria		[27]
CPXV	GRI-90	Human	1990	X94355	Moscow, Russia	B	[45]
CPXV	FIN 2000_MAN	Human	2000	HQ420893	Tohmajärvi, Finland		[27]
CPXV	HumLit08/1	Human	2008	KC813493	Vilnius, Lithuania	C	[28]
CPXV	HumAac09/1 ^§^	Human	2009	KC813508	Aachen, Germany	D	[28]
CPXV	RatAac09/1 ^§^	Rat	2009	KC813501	Aachen, Germany		[28]
CPXV	RatGer09/1 ^§^	Rat	2009	KC813503	Germering, Germany		[28]
CPXV	HumKre08/1 ^§^	Human	2008	KC813512	Krefeld, Germany		[28]
CPXV	RatKre08/2 ^§^	Rat	2008	KC813505	Krefeld, Germany		[28]
CPXV	Ratpox09 ^§^	Rat	2009	LN864565	Marl, Germany		[46]
CPXV	HumGra07/1	Human	2007	KC813510	Graz, Austria		[28]
CPXV	GER 1998/2	human	1998	HQ420897	Eckental, Germany	E1	[27]
CPXV	Germany 91-3	Human	1991	DQ437593	Munich, Germany	E2	[47]
CPXV	FM2292	Common Vole	2011	LN864566	Baden-Wuerttemberg, Germany		[46]
CPXV	MarLei07/1	Mara	2007	KC813499	Leipzig, Germany	E3	[28]
CPXV	HumLue09/1	Human	2009	KC813494	Lübeck, Germany		[28]
CPXV	GER_1990_2	Human	1990	HQ420896	Bonn, Germany		[27]
CPXV	France-01 NANCY	Human	2001	HQ420894	Nancy, France		[27]
CPXV	NOR 1995 feline	Cat	1994	KY549151	Bergen, Norway	E4	This paper
CPXV	NOR 1994_MAN	Human	1994	HQ420899	Bergen, Norway		[27]
CPXV	UK 2000 K 1639	Cat	2000	KY549148	Bristol, UK	E5	This paper
CPXV	UK 2000 K 2739	Cat	2000	KY549149	Bristol, UK		This paper
CPXV	UK pox 5/wv1	Cheetah	1972	KY549144	London, UK		This paper
CPXV	UK 2000 K 4207	Cat	2000	KY549150	Bristol, UK		This paper
CPXV	UK_Brighton Red	Human	1937	AF482758	Brighton, UK		[48]
CPXV	UK 2000 K 2984	Cat	2000	HQ420900	Bristol, UK		[27]
CPXV	UK 2000 K 428	Cat	2000	KY549145	Bristol, UK		This paper
CPXV	UK 2000 K 779	Cat	2000	KY549146	Bristol, UK	E6	This paper
CPXV	UK 2000 K 780	Cat	2000	KY549147	Bristol, UK		This paper
CPXV	GER 1980-EP4	Elephant	1980	HQ420895	Hameln, Germany		[27]
CPXV	HumPad07/1	Human	2007	KC813496	Paderborn, Germany		[28]
CPXV	GER 2002 MKY	Marmoset	2002	HQ420898	Gӧttingen, Germany	E7	[27]
CPXV	BeaBer04/1	Beaver	2004	KC813491	Berlin, Germany	E8	[28]
CPXV	CatBer07/1	Cat	2007	KC813502	Berlin, Germany		[28]
CPXV	HumBer07/1	Human	2007	KC813509	Berlin, Germany		[28]
CPXV	HumMag07/1	Human	2007	KC813495	Magdeburg, Germany		[28]
CPXV	CatPot07/1	Cat	2007	KC813506	Potsdam, Germany		[28]
CPXV	Amadeus_2015	Horse	2015	LN879483	Germany		[31]
CPXV	EleGri07/1 ^Δ^	Elephant	2007	KC813507	Grimmen, Germany	E9	[28]
CPXV	HumGri07/1 ^Δ^	Human	2007	KC813511	Grimmen, Germany		[28]
CPXV	RatHei09/1 ^‡^	Rat	2009	KC813504	Heidelberg, Germany	E10	[28]
CPXV	JagKre08/1 ^‡^	Jaguarundi	2008	KC813497	Krefeld, Germany		[28]
CPXV	JagKre08/2 ^‡^	Jaguarundi	2008	KC813498	Krefeld, Germany		[28]
CPXV	MonKre08/4 ^‡^	Mongoose	2008	KC813500	Krefeld, Germany		[28]
CPXV	HumLan08/1 ^‡^	Human	2008	KC813492	Landau, Germany		[28]
CMLV	M-96	Camel	1996	NC003391	Kazakhstan	CMLV	[49]
CMLV	CMS	Camel	1970	AY009089	Iran		[50]
ECTV	Naval	Mouse	1996	KJ563295	USA	ECTV	[51]
ECTV	Moscow	Mouse	1947	NC004105	Moscow, Russia		[52]
MPXV	Liberia_1970	Human	1970	DQ011156	Liberia	MPXV	[53]
MPXV	Zaire-96	Human	1996	NC003310	Zaire/DRC		[54]
TATV	Dahomey 1968	Gerbil	1968	NC008291	Dahomey, Benin	TATV	[47]
VACV	Rabbitpox Utrecht	Rabbit	1941	AY484669	Utrecht, The Netherlands	VACV	[55]
VACV	Horsepox_MNR-76	Horse	1976	DQ792504	Mongolia		[56]
VARV	Bangladesh1974	Human	1976	DQ441420	Bangladesh	VARV	[47]
VARV	Brazil1966	Human	1966	DQ441419	Brazil		[47]

CPXV: Cowpox virus; CMLV: Camelpox virus; ECTV: Ectromelia virus; MPXV: Monkeypox virus; TATV: Taterapox virus; VACV: Vaccinia virus; VARV: Variola virus.

## Supplementary Materials

The following are available online at www.mdpi.com/1999-4915/9/5/101/s1. Figure S1: Dataset 1 RAxML Results; Figure S2: Dataset 2 RAxML Results; Table S1: Average uncorrected p-distance bottom left, patristic distance top right for dataset 1 for all examined taxa; Table S2: Average uncorrected p-distance bottom left, patristic distance top right for dataset 2 for all examined taxa; Table S3: Average intra-lineage genetic distance within each group, calculated using uncorrected-p distance in MEGA6.
